# Surgical Management of Barium Impaction: A Case Report

**DOI:** 10.7759/cureus.61111

**Published:** 2024-05-26

**Authors:** Berkeley Sharpe, Jacob Switzer, Winn Mathews

**Affiliations:** 1 General Surgery, Edward Via College of Osteopathic Medicine, Auburn, USA; 2 General Surgery, Brookwood Baptist Health, Birmingham, USA; 3 General Surgery, Princeton Baptist Medical Center, Birmingham, USA

**Keywords:** barium sulfate, perforation, barium obstruction, barolith, barium impaction

## Abstract

Radiographic studies are used within healthcare on a routine basis to aid in the diagnosis and management of patients with a variety of health conditions. Barium sulfate is a contrast agent that may be used to enhance certain imaging studies. Although barium-contrasted studies are generally safe, they are not without risk for complications. Barium impactions, and their management, are infrequently reported in scientific literature. We present a case of a patient with barium impaction who presented at the emergency room after a fall from standing with associated symptoms of abdominal pain, weakness, and fatigue. A non-contrast computed tomography (CT) scan performed on presentation revealed the barium impaction, and initial attempts at conservative management were unsuccessful. A decompressive colonoscopy was performed without successful dissolution of the barium. Ultimately, the patient underwent exploratory laparotomy, which revealed a contained perforation of the sigmoid colon, and a successful partial colectomy with end colostomy was performed. This case study explores the surgical management of barium impaction in a comorbid patient.

## Introduction

Barium sulfate, a commonly used contrast agent, plays a crucial role in radiologic studies [1]. Its unique composition and diagnostic effectiveness expedite the diagnosis of various gastrointestinal diseases [1,2]. Barium studies, such as swallows or enemas, provide clinicians with enhanced imaging for specific anatomical areas [3]. While generally associated with minimal adverse effects, serious complications that may occur include gastrointestinal perforation, obstruction, and impaction of a barollith [4,5]. The term “barolith” is defined as a mixture of barium and stool compacting after the use of barium for radiological study [4-6]. Due to the lack of standardized treatment guidelines, physicians must be attuned to the signs and symptoms of barium complications. This case report highlights the surgical management of barium impaction after nonsurgical interventions failed [4-6].

## Case presentation

A 67-year-old female initially presented to the emergency room for severe fatigue. The patient was found to be severely anemic, and a non-contrast computed tomography (CT) revealed active colitis and esophagitis. It was during this hospitalization that the patient received a barium study to further investigate the cause of the patient's severe anemia. After appropriate fluid resuscitation and treatment of iron deficiency anemia with iron infusions, the patient was stable and discharged with antibiotics and plans for outpatient follow-up. Unfortunately, the patient sustained a fall from standing at home due to fatigue, and she presented to the emergency department again. At this time, the patient noted intense abdominal pain, with no recent bowel movement. She denied symptoms of diarrhea, nausea, vomiting, or any evidence of bleeding. The patient’s past medical history is significant. She has a history of hypertension, hyperlipidemia, chronic obstructive pulmonary disease (COPD), type 2 diabetes mellitus, schizoaffective disorder, and metastatic moderately differentiated lung adenocarcinoma undergoing chemotherapy. Abnormal vital signs and laboratory values are listed in Table 1. 

**Table 1:** Abnormal laboratory values obtained in the emergency department

Lab Test	Result	Reference Range
Pulse	115	60-100 bpm
White Blood Cell Count	28,000	4,500-11,000 cells/μL
CO_2_	16	23-29 mmol/L
BUN (Blood Urea Nitrogen)	24	7-20 mg/dL
Creatinine	2.69	0.6-1.1 mg/dL
Anion Gap	19	8-16 mmol/L
Alkaline Phosphatase	416	44-147 U/L
AST (Aspartate Transaminase)	40	5-34 U/L
ALT (Alanine Transaminase)	54	5-55 U/L
Hemoglobin	8.8	12-16 g/dL
Hematocrit	26	36-48%
Platelets	103	150-450 x 10^3^/μL
Prothrombin Time	15.6	11-13.5 seconds
INR (International Normalized Ratio)	1.48	0.8-1.2

Notably, the patient's lactic acid level was within normal limits. Non-contrast CT scans of the brain and cervical spine were obtained to evaluate for potential injury associated with her fall; these were negative for acute process and demonstrated age-appropriate changes. A chest X-ray was also performed, revealing mild atelectasis. 

**Figure 1:**
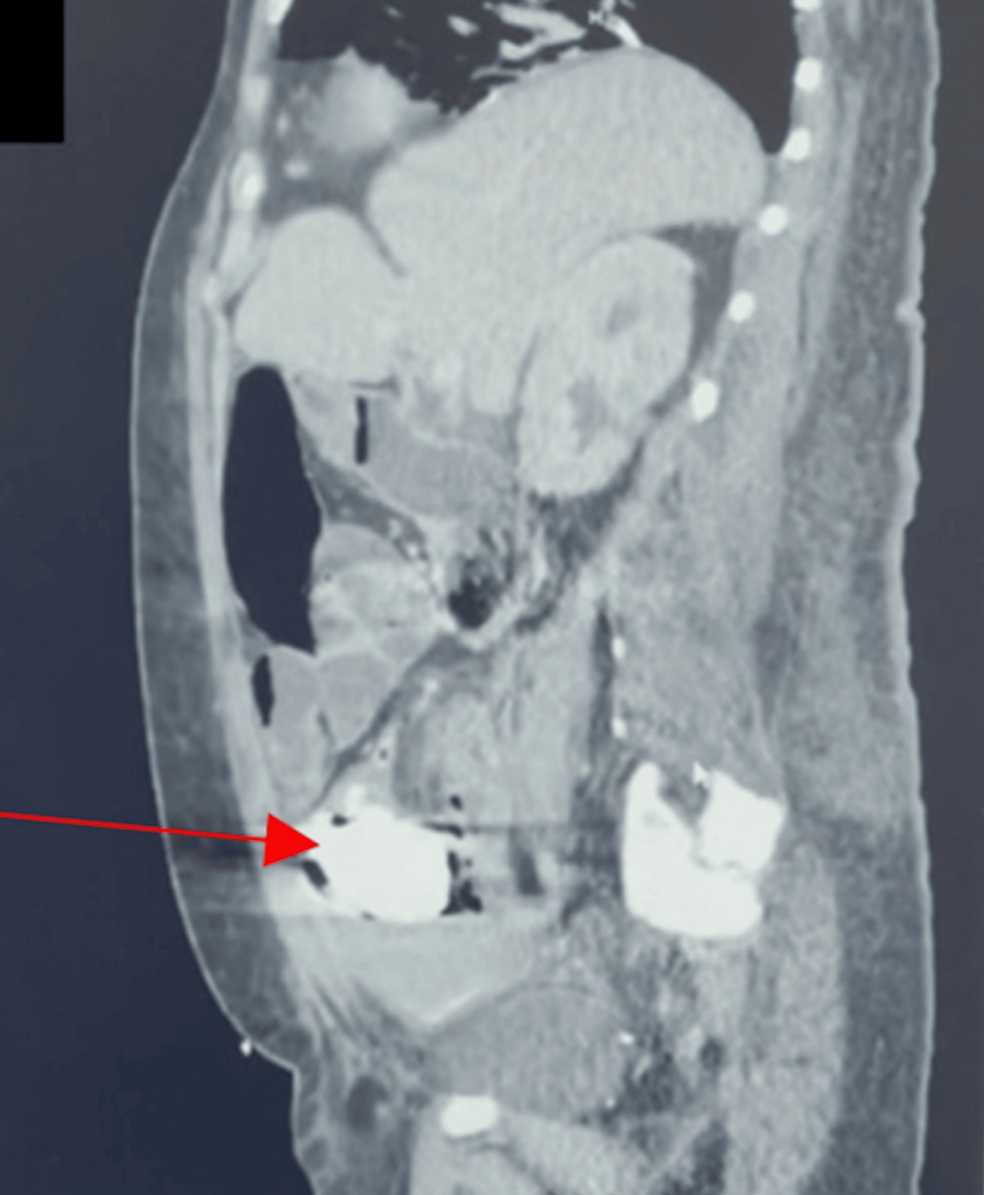
Sagittal CT of abdomen and pelvis with/without contrast showing barium impaction (red arrow) in sigmoid colon CT: Computed tomography

**Figure 2:**
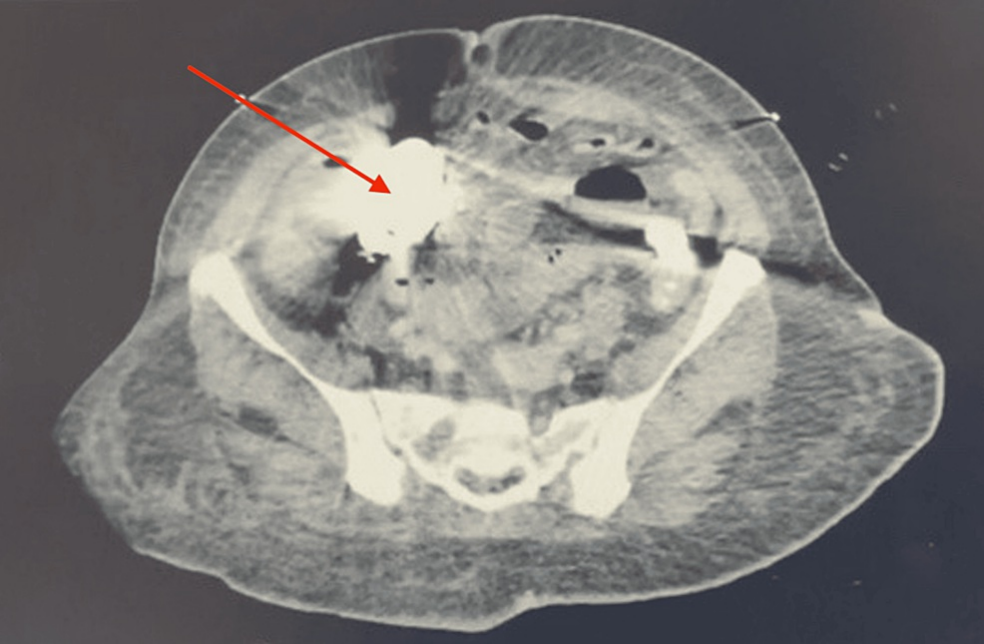
Axial CT of abdomen and pelvis with/without contrast showing barium impaction (red arrow) in sigmoid colon CT: Computed tomography

The patient was admitted to the hospitalist service, underwent intravenous (IV) fluid resuscitation, and was treated with IV meropenem (Merrem IV, Pfizer), as it was initially thought to be pseudomembranous colitis. On presentation, the patient’s abdomen was tender in all four quadrants with significant guarding in the right lower quadrant. Over the next 24 hours, the patient’s abdominal tenderness improved, but her white blood cells (WBCs) increased to 35,000 cells/uL. Non-contrast CT scans of the patient's abdomen and pelvis were obtained. The radiologist noted that “abdominopelvic evaluation is mildly limited secondary to streak artifact from barium contrast in the patient’s colon,” but no evidence of bowel perforation was present (Figures 1-2). Surgical consultation was obtained and a colonoscopy was planned for the following day to attempt endoscopic dissolution or retrieval of the barium impaction. The possible requirement of an exploratory laparotomy, depending on the success of the colonoscopy, was discussed with the patient and family. After a detailed discussion of the risks, benefits, and alternatives to the proposed procedure, the patient and family voiced understanding and elected to proceed.

The patient’s respiratory status was labile before the colonoscopy, and due to her increased risk for pulmonary complications secondary to sedation, the patient was electively intubated for the procedure. During the colonoscopy, a severe stricture was encountered in the sigmoid colon that was unable to be traversed safely despite multiple attempts at patient repositioning, applying manual pressure to the abdomen, pneumatic insufflation, and irrigation with water. An attempt using a pediatric colonoscope was discussed but ultimately decided against. After a discussion with the family regarding the incomplete colonoscopy and its findings, the family elected to proceed with exploratory laparotomy on the same day. 

The patient was transported to the operative theater where an exploratory laparotomy was performed via a midline incision. Upon entering the abdomen, abundant purulent fluid was present in the pelvis and left lower abdomen. The sigmoid colon was friable and covered with fibrinous debris at the site where a perforation had attempted to wall off. Manual palpation of the descending colon revealed impacted barium to the level of splenic flexure. After mobilization of the colon, a left descending colectomy was performed in the standard fashion; the colon was transected proximally near the splenic flexure and distally at the rectosigmoid junction to include the perforated portion of the sigmoid colon. The sigmoid perforation was confirmed by visualization upon removal of the left colon specimen. The specimen was removed and sent for pathologic evaluation. 

An end colostomy was brooked and matured in the standard fashion. Attempts were made to mobilize the ostomy, but inspissated barium was stuck on the colon wall, proving to be very difficult to remove. The patient was started on mineral oil via a nasogastric tube to aid in the dissipation of residual barium in the colon. A Gastrografin enema via the ostomy was discussed to clear out any remaining barium from her colon once the patient has improved and wound healing has progressed. 

## Discussion

The presentation for a barolith impaction can vary from mild nausea to severe abdominal pain and distention. In our patient's case and others in the literature, common presenting signs on admission were abdominal pain and an elevated white count [2, 4-10]. Our patient did reveal that she had not had a recent bowel movement, which helped direct our treatment plan. With a low risk for barium complications and nonspecific patient presentations, barolith impactions can be hard to diagnose. If a barium impaction is suspected, an abdominal X-ray should be completed to discern between any remaining barium or complete barium removal [6]. Once barium has impacted, however, the risk of morbidity and mortality increases dramatically [7]. Therefore, patients should be educated and counseled on adequate fluid intake post-barium study [8]. The use of laxatives has been reported to aid in barium clearance as routine post-barium study care as well as a conservative treatment option [7-9]. Additionally, patients should be aware to report a lack of bowel movements to their physician.

Barolith impactions are relatively rare, with Kurer et al. finding only 31 patients with a barolith impaction in a systemic review occurring from 1950-2006 [5]. It is hypothesized that elderly patients are more at risk for barium impactions due to decreased gastrointestinal transit times [8-10]. One can also assume that patients with medical conditions that affect colon motility, drug use, and anatomical changes causing narrowed colonic lumen are at an increased risk of barolith impactions [8]. Literature reports that once swallowed, barium is cleared from the colon within one week in 57% of patients, with barium completely cleared within four weeks in most patients [9]. In patients complicated with barium impaction, the mean day of bowel obstruction is three days [7]. The most common places for barium obstructions to form are the descending and rectosigmoid colon [2,9]. This can be attributed to the narrowing of the colonic lumen as it progresses to the distal end of the colon and the barolith accumulating mass as it mixes with intestinal contents [2]. The pathophysiology of barium obstructions depends on the anatomy and gut motility of each patient. 

In reviewing the recent scientific literature, nonsurgical and surgical attempts have been made to resolve barium obstructions. Nonsurgical methods reported in the literature include manual extractions, water or mineral oil enemas, laxatives, and endoscopic removal [8]. When a colonoscopy approach was used, physicians used a high-pressure jet stream or prolonged water irrigation to achieve barium dissolution [6,8]. Surgical methods differ in the presentation of each individual patient. A total or partial colectomy with colostomy is reserved for cases of colonic perforation, whereas a colotomy with barolith removal can be appropriate in non-perforated cases [8]. Management of barium obstructions should depend on the initial presentation, conservative treatment response, and patient stability. 

## Conclusions

The absence of standardized treatment guidelines for barolith obstruction poses a challenge for physicians. Understanding the risk factors, mean days of obstruction, and common sites of obstruction aids in early diagnosis and intervention. When physicians are aware of a potential impaction forming, they can intervene and provide early, conservative treatment options for patients. Further research is needed to establish clearer protocols for the management of these rare but potentially serious events in clinical practice.
